# Wide-field Ca^2+^ imaging reveals visually evoked activity in the retrosplenial area

**DOI:** 10.3389/fnmol.2015.00020

**Published:** 2015-06-08

**Authors:** Tomonari Murakami, Takashi Yoshida, Teppei Matsui, Kenichi Ohki

**Affiliations:** ^1^Department of Molecular Physiology, Graduate School of Medical Sciences, Kyushu UniversityFukuoka, Japan; ^2^CREST, Japan Science and Technology AgencyTokyo, Japan

**Keywords:** wide-field Ca^2+^ imaging, transgenic mouse, GCaMP3, visual response, retrosplenial area

## Abstract

Due to recent advances of genetic manipulation, mouse brain has become a useful model for studying brain function, which demands whole brain functional mapping techniques in the mouse brain. In the present study, to finely map visual responsive areas in the mouse brain, we combined high-resolution wide-field optical imaging with transgenic mice containing the genetically encoded Ca^2+^ indicator, GCaMP3. With the high signal amplitude of GCaMP3 expressing in excitatory neurons, this system allowed neural activity to be observed with relatively fine spatial resolution and cell-type specificity. To evaluate this system, we examined whether non-visual areas exhibited a visual response over the entire surface of the mouse hemisphere. We found that two association areas, the retrosplenial area (RS) and secondary motor/anterior cingulate area (M2/AC), were significantly responsive to drifting gratings. Examination using gratings with distinct spatiotemporal frequency parameters revealed that the RS strongly responded to high-spatial and low-temporal frequency gratings. The M2/AC exhibited a response property similar to that of the RS, though it was not statistically significant. Finally, we performed cellular imaging using two-photon microscopy to examine orientation and direction selectivity of individual neurons, and found that a minority of neurons in the RS clearly showed visual responses sharply selective for orientation and direction. These results suggest that neurons in RS encode visual information of fine spatial details in images. Thus, the present study shows the usefulness of the functional mapping method using a combination of wide-field and two-photon Ca^2+^ imaging, which allows for whole brain mapping with high spatiotemporal resolution and cell-type specificity.

## Introduction

Whole brain functional mapping, which measures brain activity using techniques such as functional magnetic resonance imaging (fMRI), is useful to exhaustively explore and define areas related to particular information processing in humans and non-human primates (Belliveau et al., 1991; Stoewer et al., 2010; Mantini et al., 2012; Park and Friston, 2013). Although these techniques can be applied to smaller animals like mice (Desai et al., 2011; Jonckers et al., 2011; Guilfoyle et al., 2013), it is difficult to finely map responsive areas in the mouse brain because the spatial resolution is insufficient to dissociate neural activity in small areas. Moreover, fMRI measures hemodynamic signal derived from neural populations including various cell-types, which makes it difficult to extract activity information from the particular cell-type.

Recently, owing to the development of imaging technique and genetic manipulation, mouse vision has been widely used as a model system to investigate detailed neural function for visual information processing. For example, previous studies using two-photon Ca^2+^ imaging have revealed spatial and functional organization of orientation selectivity in primary visual areas (V1; Ohki et al., 2005) and that neurons in higher visual areas surrounding V1 are specialized to process characteristic spatiotemporal frequency information (Andermann et al., 2011; Marshel et al., 2011; Roth et al., 2012). These visual areas send projection to many areas outside of the visual areas (Wang et al., 2011, 2012; Zingg et al., 2014). In primates, visually responsive areas, including sensory association areas such as parietal and frontal areas, has been defined (Stein and Stanford, 2008). In mice, however, it remains unclear which cortical area outside of visual areas exhibits visual response and it is important to define visual responsive areas for comprehensive understanding of mouse visual system.

In the present study, we performed wide-field Ca^2+^ imaging with transgenic mice expressing genetically encoded Ca^2+^ indicator (GECI), GCaMP3 in excitatory neurons (Zariwala et al., 2012) to map cortical areas responsive to drifting gratings over an entire surface of hemisphere. We found that two sensory association areas, retrosplenial area (RS) and secondary motor/anterior cingulate area (M2/AC), were significantly responsive to drifting gratings. RS more strongly responded to gratings with high-spatial and low-temporal frequencies (HSF–LTF) than gratings with low-spatial and high-temporal frequencies (LSF–HTF). Moreover, cellular imaging using two-photon microscopy revealed that the neurons in RS had orientation and direction selectivity. These results suggest that neurons in RS encode information about the orientation of edges and are specialized to process fine details of visual images. Thus, through these examinations, we show usefulness of functional mapping using a combination of wide-field and two-photon Ca^2+^ imaging.

## Materials and Methods

### Animal Preparation and Surgery for *In Vivo* Wide-Field Ca^2+^ Imaging

Emx1-IRES-cre (Gorski et al., 2002, Jax stock # 005628) and Ai38 (Zariwala et al., 2012, Jax stock # 014538) mice were obtained from the Jackson Laboratory. These mice were crossed to obtain transgenic mice in which all of the cortical excitatory neurons expressed GCaMP3. The transgenic mice (P60–90) were prepared for *in vivo* wide-field Ca^2+^ imaging. Anesthesia was induced and maintained during surgery with 3 and 1–2% isoflurane, respectively. During recording, mice were sedated with chlorprothixene (0.3–0.8 mg/kg, Sigma–Aldrich, St. Louis, MO, USA) and isoflurane was reduced to 0.5% (Smith and Häusser, 2010; Marshel et al., 2011; Akerboom et al., 2012). A custom-made metal head plate was attached to the skull using dental cement (Sun Medical Company, Ltd, Shiga, Japan). The skull over the cortex were kept moist for transparency, and sealed with artificial cerebrospinal fluid [ACSF; 150 mM NaCl, 2.5 mM KCl, and 10 mM HEPES (pH 7.4)] and a glass coverslip. Body temperature was maintained at 37°C using a heating pad. All experiments were carried out in accordance with the institutional animal welfare guidelines laid down by the Animal Care and Use Committee of Kyushu University, and approved by the Ethical Committee of Kyushu University.

### *In Vivo* Wide-Field Ca^2+^ Imaging

Wide-field imaging of Ca^2+^ signals *in vivo* was performed using a macro zoom fluorescence microscope (MVX-10, Olympus, Tokyo, Japan), equipped with a 1× objective (1× MVX Plan Apochromat Lens, NA 0.25, Olympus). GCaMP3 was excited by a 100 W mercury lamp through a GFP mirror unit (U-MGFPHQ/XL, Olympus; excitation peak: 488 nm, emission peak: 507 nm). Ca^2+^ signals were collected at a frame rate of 10–20 Hz using a cooled CCD camera (DS-Qi1Mc, Nikon, Tokyo, Japan) controlled by NIS-elements BR (Nikon). For recording of the entire hemisphere, a rectangular region (12 mm × 9 mm) was imaged at 320 × 240 pixels, at a focus set at 0.75 mm in depth from the top of cortical surface which firstly comes into focus.

### Animal Preparation and Surgery for *In Vivo* Two-Photon Ca^2+^ Imaging

Wild type mice (C57BL/6) were prepared for *in vivo* Ca^2+^ imaging as described previously (Ohki et al., 2005; Ohtsuki et al., 2012; Hagihara and Ohki, 2013). In brief, a custom-made metal plate was mounted on the skull, and a craniotomy was carefully performed above V1 or the RS region using stereotaxic coordinates. We dissolved 0.8 mM Oregon Green 488 BAPTA-1 AM (OGB1-AM, Life Technologies, Grand Island, NY, USA) in dimethyl sulfoxide with 20% pluronic acid and mixed it with ACSF containing 0.05 mM Alexa594 (Life Technologies) or 0.025 mM sulforhodamine 101 (SR101, Sigma–Aldrich). A glass pipette (3–5 μm tip diameter) was filled with this solution and inserted into the cortex at a depth of approximately 250 μm from the surface. The solution was pressure-ejected from the pipette (about 0.5 psi for 1 s, 10–20 times). After confirming OGB1-AM loading, the craniotomy was sealed with a coverslip. After surgery, the isoflurane concentration was reduced to 0.5% and chlorprothixene (0.3–0.5 mg/kg) was administered intramuscularly before recording experiments (Smith and Häusser, 2010; Marshel et al., 2011; Akerboom et al., 2012).

### *In Vivo* Two-Photon Ca^2+^ Imaging

Changes in Ca^2+^ fluorescence in cortical neurons were monitored using a two-photon microscope (Nikon A1MP) equipped with a mode-locked Ti:sapphire laser (MaiTai Deep See, Spectra Physics, Santa Clara, CA, USA). The excitation light was focused with a 25× objective (Nikon PlanApo, NA: 1.10). The average laser power delivered to the brain was <20 mW, depending on the depth of focus. OGB1-AM was excited at 920 nm and the emission was filtered at 517–567 nm. Images were obtained using Nikon NIS Elements software. A square region of the cortex (512 × 512 pixels, approximately 250 or 330 μm on a side) was imaged at 30 Hz frame rate. Images were obtained from depths of 200–300 μm for experiments in layers 2/3. Stereotaxic coordinates of recording sites were 1–1.5 mm anterior from lambda, 2.5–3.0 mm lateral from the midline for V1, 2.0–2.5 mm anterior from lambda and approximately 0.5 mm lateral from midline for the RS which corresponded to the dysgranular area of the RS (Franklin and Paxinos, 2008).

### Visual Stimulation

The visual stimulus sets were generated using custom-written programs running on PsychoPy (Peirce, 2007). Stimulus presentation was synchronized with frame acquisition of images using a counter board (NI USB-6501, National instruments, Austin, TX, USA). We positioned a 32-inch LCD monitor 18 cm from the mouse’s right eye and the monitor was adjusted to be parallel to the mouse’s right eye. In wide-field imaging, drifting sinusoidal-wave gratings (100% contrast) were presented in an aperture (40° diameter). The three parameter sets of SF and TF were used ([SF, TF] = [0.08, 1.0], [0.04, 2.0], [0.01, 4.0], [cycles per degree (cpd), Hz]). Each stimulus started with a blank period of uniform gray (6 s), which was followed by 2 s visual stimulation. The stimuli were presented in random order and repeated 50 times. In two-photon imaging, drifting square-wave gratings (100% contrast) were presented in full screen. The TF was set at 1.0–4.0 Hz and the SF was set at 0.01–0.16 cpd for the RS recording. The SF and TF were set at 2.0 Hz and 0.04 cpd for V1 recording. Each stimulus started with a blank period of uniform gray (4 s), which was followed by 4 s visual stimulation. The eight direction stimuli were presented in random order and repeated 10–15 times.

### Data Analysis

Images were analyzed with Matlab (Mathworks, Natick, MA, USA) and ImageJ (National Institutes of Health, USA). In wide-field imaging experiments, images were realigned by maximizing the correlation between frames. The baseline signal (F) of each trial was average of Ca^2+^ signals during 1 s before each stimulus onset. Visual response signal (dF) of each trial was average of Ca^2+^ signals during each stimulus period. To obtain response map, we calculated the fluorescence ratio change (dF/F) map of each trial, and averaged them across trials. The *T*-value map was calculated across the dF/F maps of 50 trials. These maps were spatially smoothed with a Gaussian filter (size: 25 × 25 pixels; sigma: 5 pixels). To define regions of interest (ROIs), six cortical regions [V1, RS, M2/AC, primary auditory area (A1), primary somatosensory area (S1), and primary motor area (M1)] were selected by reference to stereotaxic coordinates in the Mouse Brain Atlas (Franklin and Paxinos, 2008). ROI size was a 7 × 7 pixel square (about 260 μm × 260μm). Time courses of individual ROIs were extracted by summing pixel values within ROIs. Time course of each trial was normalized by F of each trial. We defined the initial rise of response to visual stimuli as the time point when calcium signal exceeded the threshold (three SD of the baseline signal).

For pixel-based analysis in two-photon imaging experiments, images were averaged over stimulus repetitions and stimulus period. dF maps were obtained by subtracting the averaged image of the blank period from that of each stimulus (total eight directions). Color-coded direction and orientation maps were obtained from these eight direction dF maps and four orientation dF maps which were average of the two opposite direction dF maps. The hue of each pixel was determined by the preferred direction and orientation defined by vector averaging, the color saturation was proportional to [1–circular variance (CV)] of the direction and orientation, and the brightness was proportional to the F to the best direction and orientation. Color-coded maps were spatially smoothed with a Gaussian filter (size: 5 × 5 pixels; sigma: 1 pixel).

For cell-based analysis in two-photon imaging experiments, ROIs of individual neurons were automatically identified by template matching with a circular template about the size of the soma. Automatically identified neurons were visually inspected, and errors were corrected manually. Time courses of individual neurons were extracted by summing pixel values within cell contours. Slow drift of the baseline signal over minutes was removed by a low-cut filter (Gaussian, cut-off, 2 minutes). High frequency noise was removed by a high-cut filter [third order Savitzky–Golay filter with 31 time points (∼1 second)] only for time course presentation. To minimize neuropil signal contamination, the time course of the neuropil signal obtained from surrounding part of a cell body was subtracted from each neuron’s time course after multiplying by a scaling factor (Kerlin et al., 2010). The scaling factor was determined in each field of view (FOV) by computing the ratio of the fluorescent signal of the blood vessel to that of the surrounding background signal averaged across several blood vessels. After removing the neuropil signal, the time course was used to obtain the signal change (mean fluorescence change normalized by baseline, dF/F) in response to each stimulus direction/orientation. Visually responsive neurons were defined by the following criteria; *p* < 0.01 by analysis of variance (ANOVA) applied to dF/F across blank and eight direction periods and dF/F > 2% in response to the best direction. Of all responsive neurons, direction-selective neurons were defined by ANOVA (*p* < 0.01) across eight directions and direction selectivity index (DSI > 0.33, see below). Orientation-selective neurons were defined by ANOVA (*p* < 0.01) across four orientations and orientation selectivity index (OSI > 0.33, see below). In the analysis of the RS, in each FOV, we chose a single spatial and temporal frequency parameter set in which the largest number of responsive neurons was obtained, and used data of this parameter for further analyses.

For responsive neurons, DSI and OSI were defined as follows: DSI, (response to preferred direction – response to null direction)/(response to preferred direction + response to null direction); OSI, (response to preferred orientation - response to orthogonal orientation)/(response to preferred orientation + response to orthogonal orientation). Direction and orientation selectivities were also assessed by (1-CV) of direction and orientation, respectively.

### Detection of GCaMP3 Expression of Neurons and Astrocytes

GCaMP3 expression of neurons and astrocytes of a Emx1-GCaMP3 mouse was recorded using two-photon microscope. To label astrocytes, SR101 was injected to layer 2/3. We imaged three planes at the depth of 180–260 μm. ROIs of individual neurons and astrocytes were manually identified by cropping around SR101 negative and positive cells, respectively (*n* = 15 cells each from three planes). GCaMP3 localized at cytosol with excluding nucleus. Therefore, the GCaMP3 intensities of individual cells were determined as the top 10% intensities of all pixels in each ROI. ROIs of blood vessels running horizontally were manually identified, and the background intensity in each plane was defined as GCaMP3 intensity averaged across vessel ROIs. The background intensity was subtracted from the GCaMP3 intensities of cells in the same plane. The subtracted intensities of cells were normalized by averaged intensities of SR101 negative cells (neurons) in each plane.

### Statistical Analyses

All data are presented as the mean ± SEM, unless stated otherwise. Visually responsive ROIs of wide-field imaging were defined by Student’s *t*-test (*p* < 0.05). An ANOVA was performed when more than two groups were compared, which was followed by Tukey’s honestly significant difference (HSD) test. Throughout the study, *p* < 0.05 was considered statistically significant, other than the definitions of visually responsive ROIs and selective neurons. In wide-field imaging experiments, the sample size n was defined as the number of animals. In two-photon imaging experiments, the sample size *n* was defined as the number of neurons.

## Results

### RS and M2/AC were Responsive to Drifting Gratings

**FIGURE 1:**
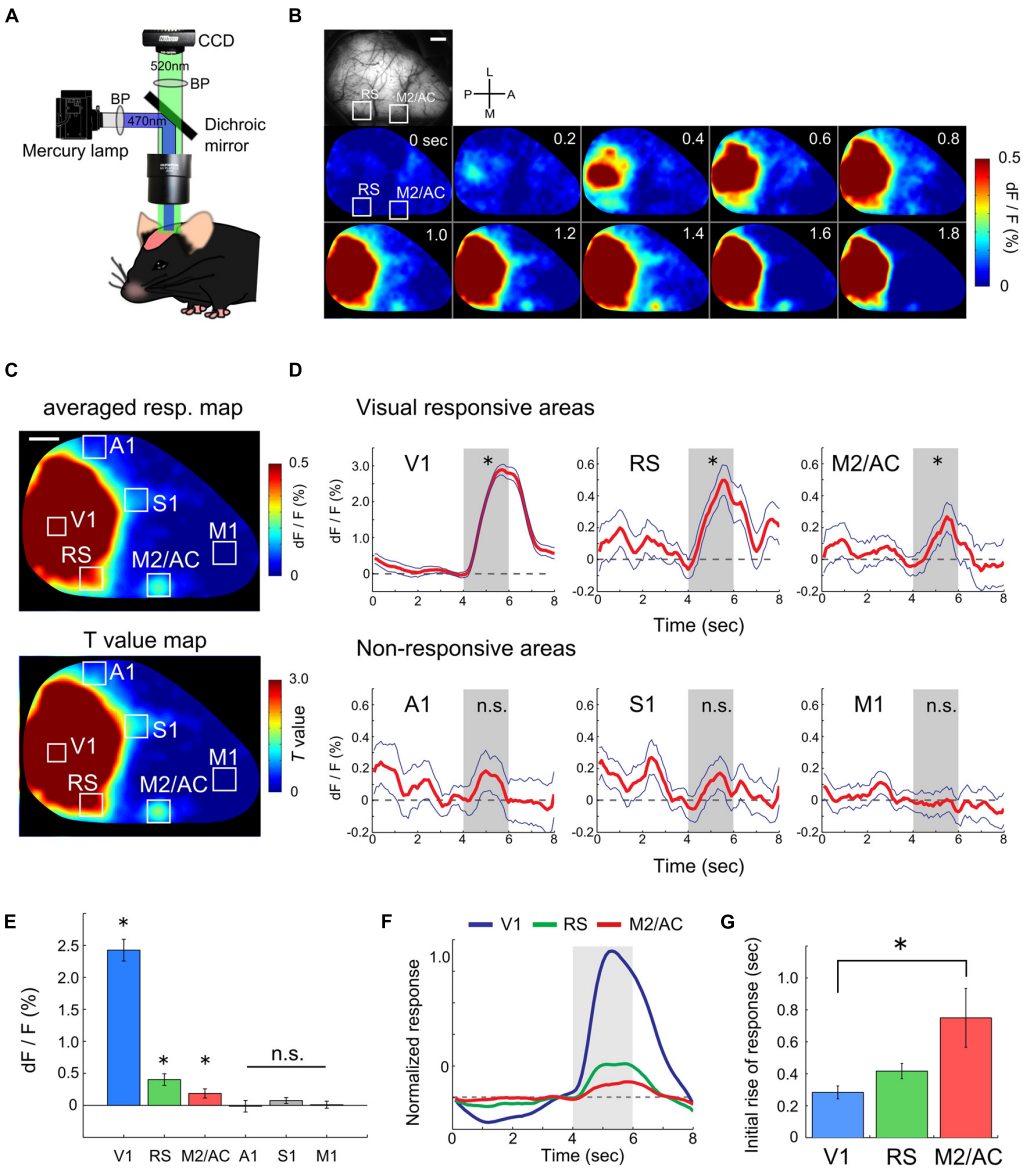
**Functional mapping of visual responsive areas. (A)** Experimental setup of wide-field Ca^2+^ imaging. **(B)** An example of a visual response in an entire hemisphere. (Top-left) Shows field of view (FOV). White two squares indicate regions of the RS and M2/AC. From upper left to lower right, sequential Ca^2+^ imaging snapshots from stimulus onset are presented (frame rate at 200 ms). **(C)** Representative response map averaged across 50 trials (top) and corresponding *T*-value map (bottom). Same animal as in **(B)**. **(D)** Representative average time courses of visually responsive (upper) and non-responsive areas (bottom). Each time course was obtained from regions of interests (ROIs) located in each area. ROI size was 260 μm × 260 μm which smaller than the white squares in **(B)** and **(C)**. Blue line: SE. Dashed line: baseline of Ca^2+^ signal. Gray shade: stimulus period. **(E)** Averaged signal change (dF/F) across animals (*n* = 6 mice). **(F)** Averaged time courses of the visual response in V1, RS, and M2/AC across animals (*n* = 6 mice). **(G)** Timing of the initial rises of the visual response. Error bar: SE, ^∗^*p* < 0.05, n.s.: non-significant, by Student’s *t*-test in **(D)** and **(E)**, and by ANOVA and *post hoc* Tukey’s HSD test in **(G)**. Scale bars in **(B)** and **(C)**: 1 mm.

To evaluate the ability of functional mapping by wide-field Ca^2+^ imaging in GECI transgenic mice, we explored visually responsive areas over the entire hemisphere including areas outside of the visual cortices. We recorded transcranial Ca^2+^ signals from Emx1-GCaMP3 mice (Zariwala et al., 2012) to monitor the visual response to drifting gratings (0.02 cpd, 1.0 Hz) under light anesthesia (**Figure 1A**). Because cre expression is observed in both glial cells and excitatory neurons in the Emx1-IRES-cre line (Gorski et al., 2002), we checked the GCaMP expression in astrocytes with injection of SR101 into Emx1-GCaMP3 mice (Supplementary Figure S1). Compared to GCaMP3 expression of neurons at the cytosol, we observed significantly weaker GCaMP3 expression in SR101 positive astrocytes (Supplementary Figure S1F). Moreover, recent studies reported that astrocytes do not respond to grating stimuli in mouse visual cortex (Bonder and McCarthy, 2014; Paukert et al., 2014). Therefore, we considered that the GCaMP3 signals of astrocytes were negligibly small. **Figures 1B–D** show an example of recorded transcranial visual response. Apparent visual responses were observed in the V1 and lateral and medial higher visual areas (**Figures 1B,C**). Outside of the visual areas, we found that the RS and M2/AC responded to the gratings. Visual responses in these areas were significant (**Figures 1B–D**, V1: *p* < 10^-26^, RS: *p* < 10^-5^, M2/AC: *p* < 0.01; Student’s *t*-test), though the response amplitudes in the RS and M2/AC were smaller than those in visual areas. A significant response was not observed in other areas (**Figure 1D**, A1: *p* = 0.155, S1: *p* = 0.253, M1: *p* = 0.733; Student’s *t*-test). Population analysis of the response amplitudes across six mice confirmed that the V1, RS and M2/AC were significantly responsive to gratings (**Figure 1E**, V1: *p* < 10^-4^, RS: *p* < 0.007, M2/AC: *p* < 0.047), but other areas were not (A1: *p* = 0.893, S1: *p* = 0.166, M1: *p* = 0.859; Student’s *t*-test). Because the response in V1 seemed to begin earlier than those in the RS and M2/AC in the representative animal (**Figure 1B**), we compared the initial rise of the visual responses among these three areas. The initial rise of response in the RS and M2/AC were delayed compared with V1, and the difference between V1 and M2/AC was statistically significant across animals (**Figures 1F,G**, *p* < 0.05; ANOVA and *post hoc* Tukey’s HSD test). Taken together, these results reveal that the RS and M2/AC respond to drifting gratings with latency comparable to that in V1.

### Visual Response Properties of the RS and M2/AC

**FIGURE 2:**
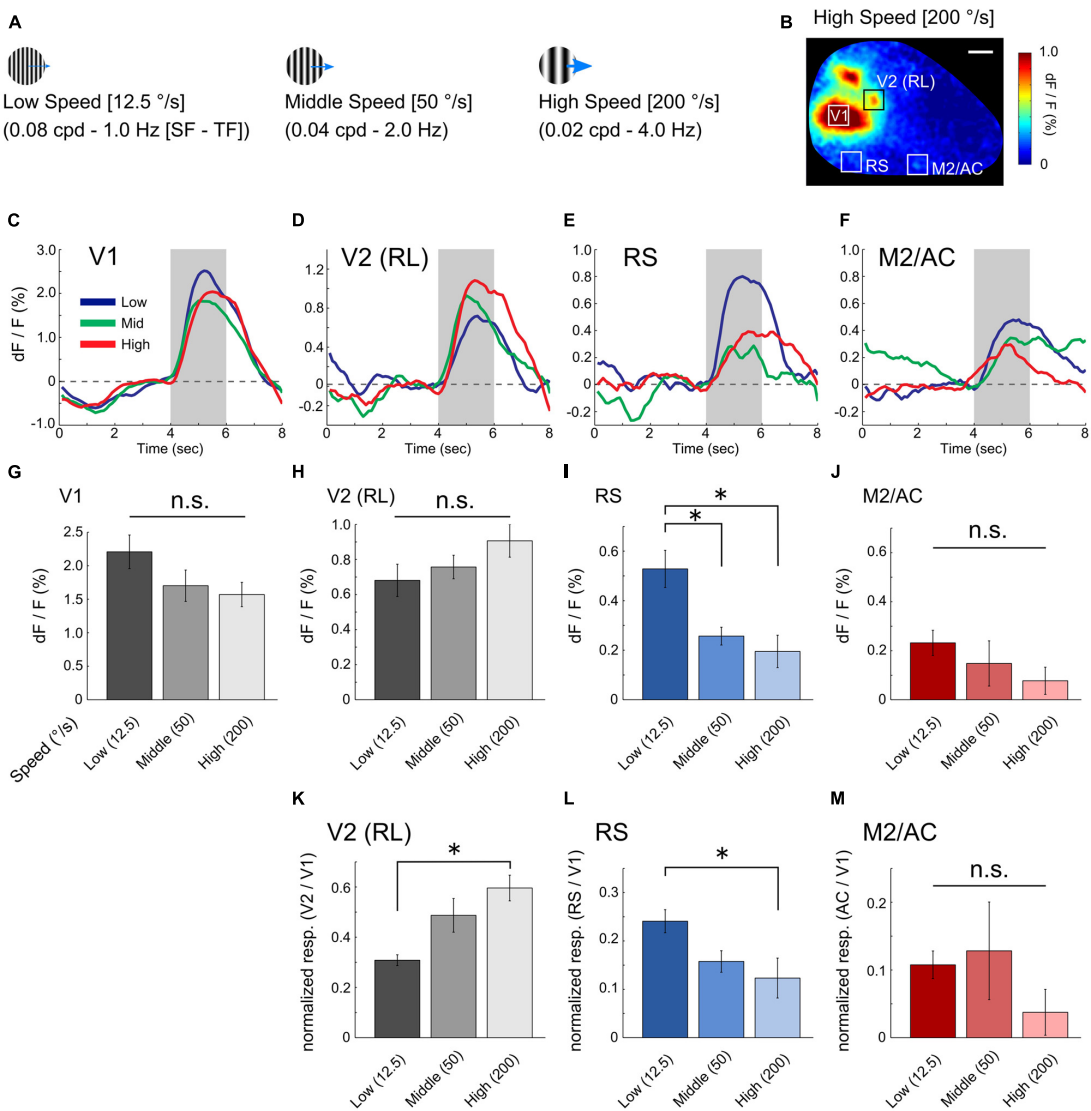
**Visual response properties in V1, RS, and M2/AC. (A)** Schematics of drifting gratings with three spatiotemporal frequency parameters. **(B)** An example of the visual response to high speed gratings. **(C–F)** Averaged time courses of V1, V2 (RL), RS, and M2/AC in the representative animal. **(G–J)** Averaged signal changes in visual response to three gratings in V1 (**G**, from left to right, 2.208 ± 0.251%, 1.701 ± 0.234%, 1.570 ± 0.181%), RL (**H**, 0.680 ± 0.092%, 0.757 ± 0.067%, 0.906 ± 0.093%), RS (**I**, 0.528 ± 0.075%, 0.257 ± 0.036%, 0.195 ± 0.066%), and M2/AC (**J**, 0.232 ± 0.051%, 0.148 ± 0.092%, 0.077 ± 0.055%). **(K–M)** Normalized response in the RL (**K**, 0.308 ± 0.022, 0.487 ± 0.067, 0.596 ± 0.051), RS (**L**, 0.241 ± 0.023, 0.158 ± 0.022, 0.123 ± 0.041), and M2/AC (**M**, 0.108 ± 0.020, 0.128 ± 0.072, 0.03 ± 0.034). Averaged across animals (*n* = 6 mice). Error bar: SE, ^∗^*p* < 0.05, n.s.: non-significant by ANOVA and *post hoc* Tukey’s HSD test. Scale bar in **(B)**: 1 mm.

We next investigated the visual response properties of the RS and M2/AC. Previous studies in mice have reported that low speed gratings (12.5 °/s) with high spatial and low temporal frequency [HSF–LTF; (0.08 cpd, 1.0 Hz)] evoke visual responses in a set of visual areas belonging to the ventral stream, and high speed gratings (200 °/s) with low spatial and high temporal frequency [LSF-HTF; (0.02 cpd, 4.0 Hz)] evoke visual responses in another set of areas belonging to the dorsal stream (Andermann et al., 2011; Marshel et al., 2011). Thus, we used three grating stimuli with distinct SF and TF ([cpd, Hz] = [0.08, 1.0], [0.04, 2.0] and [0.02, 4.0]; Speeds of these gratings were 12.5, 50 and 200 °/s, respectively; **Figure 2A**) to examine whether the RS and M2/AC exhibit response properties similar to those of the visual areas belonging to the ventral or dorsal streams.

This analysis revealed distinct response properties between V1 and the two association areas. V1 strongly responded to gratings of all three sets of parameters with slight preference to low speed (**Figure 2C**; averaged time courses of representative animal). The RS exhibited a stronger response to low speed gratings than the others (**Figure 2E**). The M2/AC also showed slightly higher response to low speed gratings; however, the response amplitude was small (**Figure 2F**). As an example of dorsal stream in mouse extrastriate cortex, we examined visual response in rostrolateral area (RL), which was identified as a discrete region separated from V1 based on visual response during stimulus period (**Figures 1B and 2B**). As with a previous report (Marshel et al., 2011), the RL showed higher response to high speed gratings (**Figure 2D**).

Population analysis across six animals showed that the visual response in the RS was significantly stronger following low speed grating when compared with other gratings (**Figure 2I**, *p* < 0.05; ANOVA and *post hoc* Tukey’s HSD test). The M2/AC also showed a stronger response as the speed of gratings became slower, but the effect was not significant (**Figure 2J**, *p* = 0.309; ANOVA). The RL showed a stronger response as the speed of gratings became higher, but the effect was not significant (**Figure 2H**, *p* = 0.196; ANOVA). In V1, there was no significant difference between the responses to three gratings (**Figure 2G**, *p* = 0.139; ANOVA), although V1 had a slight tendency to respond strongly to low speed gratings, similar to the RS and M2/AC. To examine whether the preference for low speed gratings in the RS and M2/AC was stronger than that in V1, we normalized the responses in the RS and M2/AC by that in V1 for each stimulus condition (**Figures 2L,M**). In the RS, the normalized visual response to low speed gratings remained stronger than the response to high speed gratings (*p* < 0.05; ANOVA and *post hoc* Tukey’s HSD test). In M2/AC, the normalized visual response to low speed and middle speed gratings seemed stronger than the response to high speed gratings, but the effect was not significant (*p* = 0.38; ANOVA). In RL, the normalized response to high speed gratings was stronger than that to low speed gratings (**Figure 2K**, *p* < 0.05; ANOVA and *post hoc* Tukey’s HSD test). Taken together, these results suggest that the RS contains characteristic functional features, distinct from V1, and similar to visual areas belonging to the ventral stream (Marshel et al., 2011).

### Orientation and Direction Selectivity of Neurons in the RS

**FIGURE 3:**
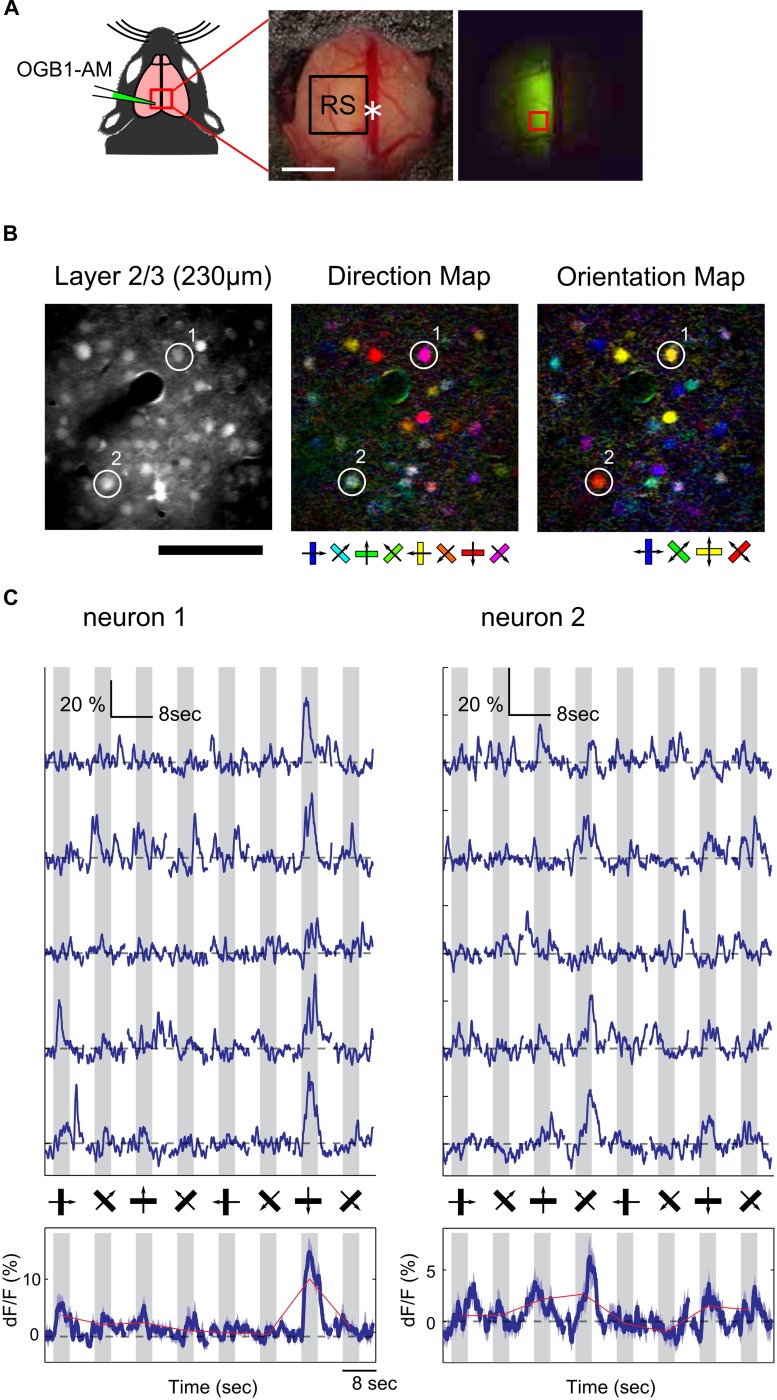
**Two-photon Ca^2+^ imaging in the RS. (A)** Schematic of OGB1-AM injection in the RS. White asterisk: landmark position of the RS (2.5 mm anterior from lambda). Scale bar, 1 mm. (Right) An example of an OGB1-AM injected region. Red square: imaging FOV shown in **(B)**. **(B)** Representative FOV (left) and color-coded maps of preferred direction (center) and orientation (right) of neurons in the RS. Scale bar: 100 μm. **(C)** Representative time courses of two neurons shown in **(B)**. (Upper) Examples of five trials. (Bottom) Averaged time courses from 15 trials. Dashed line: baseline of Ca^2+^ signal. Gray shade: stimulus period.

We found that the RS was responsive to visual stimulation, but it was still unknown whether information associated with the direction and orientation of the drifting gratings was transmitted to the RS. Because wide-field Ca^2+^ imaging lacks cellular resolution, we could not examine orientation and direction selectivity of individual neurons. Thus, we recorded neural activity at the cellular level using two-photon Ca^2+^ imaging to examine orientation and direction selectivity of neurons in the RS. Unfortunately, fluorescent signals from Emx1-GCaMP3 mice were not bright enough for cellular imaging, thus the synthetic Ca^2+^ indicator (OGB1-AM) was injected in the RS (**Figure 3A**; 2.0–2.5 mm anterior from lambda and 0.5–1.0 mm left from the central suture) of wild-type mice and the visual response of neurons loaded with the indicator was monitored. **Figure 3B** shows examples of orientation and direction maps. Ca^2+^ signal time courses of two representative neurons clearly showed a selective response to gratings drifting in a particular direction (**Figure 3C**).

**FIGURE 4:**
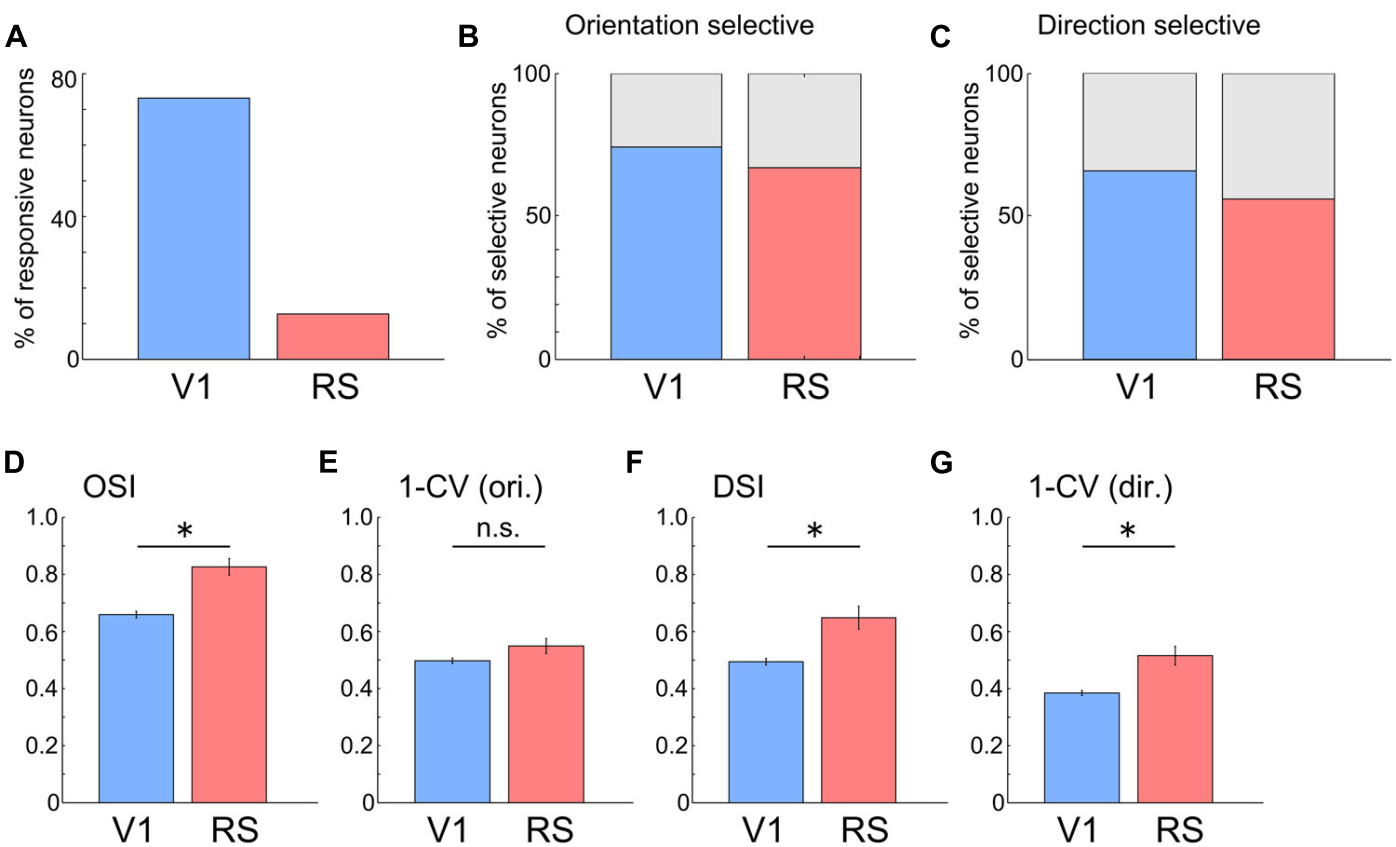
**Orientation and direction selectivity in the RS. (A)** Proportion of responsive neurons to all recorded neurons. V1: 73.1%, 518/708 neurons; RS: 12.7%, 82/645 neurons. **(B)** Proportion of orientation selective neurons to all responsive neurons. V1: 74.3%. RS: 67.1%. **(C)** Proportion of direction selective neurons to all responsive neurons. V1: 65.8%. RS: 56.1%. **(D–G)** Comparison of OSI (**D**, V1: 0.66 ± 0.01, RS: 0.83 ± 0.03), 1-CV (orientation) (**E**, V1: 0.50 ± 0.01, RS: 0.55 ± 0.03), DSI (**F**, V1: 0.49 ± 0.01, RS: 0.65 ± 0.04) and 1-CV (direction) (**G**, V1: 0.38 ± 0.01, RS: 0.52 ± 0.03) between neurons in V1 and RS. ^∗^*p* < 0.05, n.s.: non-significant by Wilcoxon’s rank-sum test.

Next, we compared the response properties of neurons in the RS with those in V1 (RS: *n* = 645 neurons from three mice; V1: *n* = 708 neurons from two mice). Compared with neurons in V1, the proportion of visually responsive neurons in the RS was very low (** Figure 4A**, V1: 73.1%, 518/708 neurons; RS: 12.7%, 82/645 neurons). However, many of the responsive neurons in the RS were orientation and direction selective, (**Figures 4B,C**; Direction selective neurons: 56.1%; Orientation selective neurons: 67.1%), at a proportion comparable to those in V1 (Direction selective neurons: 65.8%; Orientation selective neurons: 74.3%). OSI, DSI and 1–CV (direction) in the RS were higher than those in V1[**Figures 4D,F,G**; OSI, V1: 0.66 ± 0.01, RS: 0.83 ± 0.03, *p* < 10^-7^; DSI, V1: 0.49 ± 0.01, RS: 0.65 ± 0.04,*p* = 10^-4^; 1–CV (direction), V1: 0.38 ± 0.01, RS: 0.52 ± 0.03, *p* < 0.001; Wilcoxon’s rank-sum test]. However, there was no significant difference in 1–CV (orientation) between the RS and V1 (**Figure 4E**, V1: 0.50 ± 0.01, RS: 0.55 ± 0.03; *p* = 0.103). These results suggest that neurons in the RS encode information associated with the orientation and direction of the edges.

## Discussion

In the present study, we performed whole brain functional mapping of visual responsive areas using high-resolution wide-field optical imaging with transgenic mice expressing GCaMP3. We found that two association areas, the RS and M2/AC, were significantly responsive to drifting gratings. Moreover, the RS preferred low speed gratings with HSF–LTF. Finally, we performed cellular imaging using two-photon microscopy to examine whether neurons in RS were selective for direction and orientation of the gratings. These results indicate that neurons in the RS encode information associated with the orientation of edges and are specialized to process fine details in images rather than fast movement. Thus, the present study reveals the usefulness of wide-field Ca^2+^ imaging in GECI transgenic mice for whole brain functional mapping.

### Advantages of Wide-Field Ca^2+^ Imaging in GECI Transgenic Mice

Wide-field Ca^2+^ imaging in GCaMP transgenic mice is a useful method owing to some advantages for functional mapping. One of the advantages is the ability to monitor cell-type specific activity. For example, expression of GCaMP3 in Ai38 mice can be controlled by the cell type-specific Cre-driver mouse lines (Madisen et al., 2010; Gerfen et al., 2013). In our study, we monitored activity of excitatory neurons, using Emx1-GCaMP3 mice. Unlike fMRI, intrinsic signal optical imaging and voltage sensitive dye (VSD) imaging, which record hemodynamic or VSD signals reflecting population activity, most likely derived from all cell-types, wide-field Ca^2+^ imaging in the cell-type specific transgenic mouse lines is a convenient way to overcome this point.

Another advantage is that the Ca^2+^ signal amplitude of GCaMP is stronger than the intrinsic signals of hemodynamics and flavoproteins (Vanni and Murphy, 2014). Although intrinsic optical imaging of both hemodynamics and flavoprotein signals are widely used for functional mapping (Kalatsky and Stryker, 2003; Tohmi et al., 2006), the strong signal amplitude of GCaMP should be useful for reliable mapping. Indeed, we noticed that wide-field Ca^2+^ imaging in GCaMP3 transgenic mice is more efficient for retinotopy mapping across visual areas than intrinsic optical imaging (unpublished observation). Moreover, in the present study, we could detect weak activity of the RS and M2/AC to grating, most likely because of the strong signal amplitude of GCaMP3 (**Figure 1**). Indeed, we could not detect visual response in RS or M2/AC using intrinsic signal imaging with wild-type mice under the same anesthetic condition.

Moreover, wide-field imaging in GCaMP transgenic mice can be followed by two-photon imaging for cellular activity observation in the same animal. In a previous study, combination of wide-field and two-photon imaging revealed cellular-scale spatial distributions of auditory stimulus tuning in an area-scale functional map in A1 of GCaMP3 transgenic mice (Issa et al., 2014). Unfortunately, in the present study, imaging of the RS in Emx1-GCaMP3 mice with two-photon microscopy showed visual responses only in a small number of neurons, which was insufficient for population analysis (Supplementary Figure S2). In V1, we detected more responsive neurons by two-photon imaging in Emx1-GCaMP3 mice than in the RS. The reason why we could record more responsive neurons using OGB1-AM than GCaMP3 is probably because OGB1-AM is more sensitive to few action potentials than GCaMP3 (Akerboom et al., 2012). Recently reported improved GCaMP subtypes and their transgenic lines (Chen et al., 2013; Madisen et al., 2015) could be applicable to multi-scale imaging even in higher association areas. Taken together, wide-field Ca^2+^ imaging in GECI transgenic mice is a promising new tool for functional mapping of cortical areas.

### Visual Response in the RS and M2/AC

In mouse, several higher visual areas surrounding V1 are specialized to process characteristic spatiotemporal frequency information (Andermann et al., 2011; Marshel et al., 2011; Roth et al., 2012). These areas are considered to constitute two visual pathways corresponding to the dorsal and ventral pathways in primates (Ungerleider and Mishkin, 1982; Felleman and Van Essen, 1991). The RS and M2/AC receive projections from these higher visual areas (Wang et al., 2011, 2012; Zingg et al., 2014), and there are reciprocal connections between the RS and M2/AC (Oh et al., 2014; Zingg et al., 2014). Optogenetic and electrical stimulations of V1 and higher visual areas support the connections from the visual areas to the RS and M2/AC (Lim et al., 2012; Hishida et al., 2014). A previous study (Mohajerani et al., 2013) using VSD imaging has shown that VSD signal is restricted within the RS, M2/AC and visual areas only during short time window after flashed LED stimulation (around 55 ms after stimulation), and then quickly spreads over the entire cortex (>60 ms after stimulation). This is probably because VSD signal reflects both sub- and supra-threshold activity, while Ca^2+^ signal reported by GCaMP3 detects mostly supra-threshold activity, suitable for detecting the visually responsive areas which show supra-threshold response to visual stimulation. Further, visual response properties of the RS such as orientation, direction, spatial frequency, and temporal frequency selectivity remained unknown. To the best of our knowledge, the present study is the first to examine and characterize the visual response properties of the RS.

The RS of mice receives projection mainly from three visual areas, the posteromedial area (PM), anteromedial area (AM), and anterior higher visual area (A; Wang et al., 2012). These three areas exhibit distinct visual response properties. The previous three studies using two-photon imaging have reported that neurons in the PM prefer low speed gratings with HSF-LTF (Andermann et al., 2011; Marshel et al., 2011; Roth et al., 2012). In contrast, the majority of neurons in the AM are specialized to process high speed gratings with LSF–HTF, whereas the minority of neurons are selective to HSF–LTF (Marshel et al., 2011). Similarly, it was reported that neurons in area A were selective to high speed gratings (Gao et al., 2007). These studies suggest that AM and A belong to dorsal stream. Thus, among the three areas, the selectivity of the PM is similar to that of the RS, which suggests that visual responses of the RS are derived mostly from the PM, rather than the other two areas. Another possibility is that neurons in the AM and A projecting to the RS may be the minority of neurons that have selectivity to HSF–LTF (Glickfeld et al., 2013; Matsui and Ohki, 2013).

As for the functional properties, PM (Andermann et al., 2011; Marshel et al., 2011; Roth et al., 2012) and RS (present study) prefers low speed gratings, as other V2 areas belonging to ventral stream. These studies may suggest that RS and PM have a similar property of the ventral stream. However, PM and RS are thought to belong to dorsal stream from their areal location and inter-areal connections (Wang et al., 2012). Moreover, psychophysical and lesion studies of PM suggest that PM is important for optomotor learning (Prusky et al., 2008; Umino et al., 2008), and RS is also considered important for spatial navigation and spatial working memory (Vann et al., 2009; Kravitz et al., 2011; Yoder et al., 2011). These studies suggest that RS and PM are part of the dorsal stream (Glickfeld et al., 2014). Thus, it is more likely that the dorsal stream may contain two distinct pathways to process both inputs of high and low speed gratings.

Visual information processing in the RS is considered important for spatial navigation and spatial working memory (Vann et al., 2009; Kravitz et al., 2011; Yoder et al., 2011). The RS has head direction neurons, which become active when the mouse turns in a particular direction (Cho and Sharp, 2001). These studies also suggest that RS belong to the dorsal stream. Moreover, the RS encodes and stores memory of the landmark in the water maze task (Czajkowski et al., 2014). It is interesting to know whether and how the direction/orientation tunings observed here interact with information processing involving head direction selectivity and spatial navigation. Recently, the virtual reality system has been used to investigate the neural function of spatial navigation (Dombeck et al., 2010). A combination of virtual reality and wide-field and two-photon Ca^2+^ imaging may be a strong approach to address this question.

The M2/AC exhibited a visual response, although its visual response property was ambiguous in contrast with the RS, which was most likely due to low signal amplitude of the M2/AC (**Figures 2F,J**). Anatomically, the M2/AC receives strong projections from the RS (Oh et al., 2014; Zingg et al., 2014). It was also reported that the M2/AC is activated by electrical stimulation of the RS more strongly than that of higher visual areas (Hishida et al., 2014). These reports suggest that the M2/AC is a downstream area of the RS. Previous studies in mice suggest that the M2/AC may be important for modulating visual information processing. It was reported that neurons in the M2/AC send feedback projection to GABAergic neurons in V1 to improve visual discrimination (Zhang et al., 2014). Moreover, feedback projection from the M2/AC to higher visual areas is stronger than that to V1 (Zhang et al., 2014; Zingg et al., 2014). Thus, though these studies suggest that M2/AC is important for modulation of visual processing, details of the functional properties of the M2/AC neurons remain unknown. A combination of behavioral tasks that require feedback projection and wide-field and two-photon Ca^2+^ imaging may enable us to investigate how the M2/AC modulates neural activities in visual areas.

## Author Contributions

T. Murakami, TY, and KO designed the research. T. Murakami performed wide-field Ca^2+^ imaging experiments and analyzed the data. TY performed two-photon imaging experiments and analyzed the data. T. Murakami, TY, T. Matsui, and KO wrote the manuscript. All authors discussed the results and commented on the manuscript.

## Conflict of Interest Statement

The authors declare that the research was conducted in the absence of any commercial or financial relationships that could be construed as a potential conflict of interest.
