# Supplementary figures and images for: Wide-field Ca2+ imaging reveals visually evoked activity in the retrosplenial area
Source: Front Mol Neurosci. 2015 Jun 8;8:20. doi: 10.3389/fnmol.2015.00020 (PMC4458613; doi:10.3389/fnmol.2015.00020)

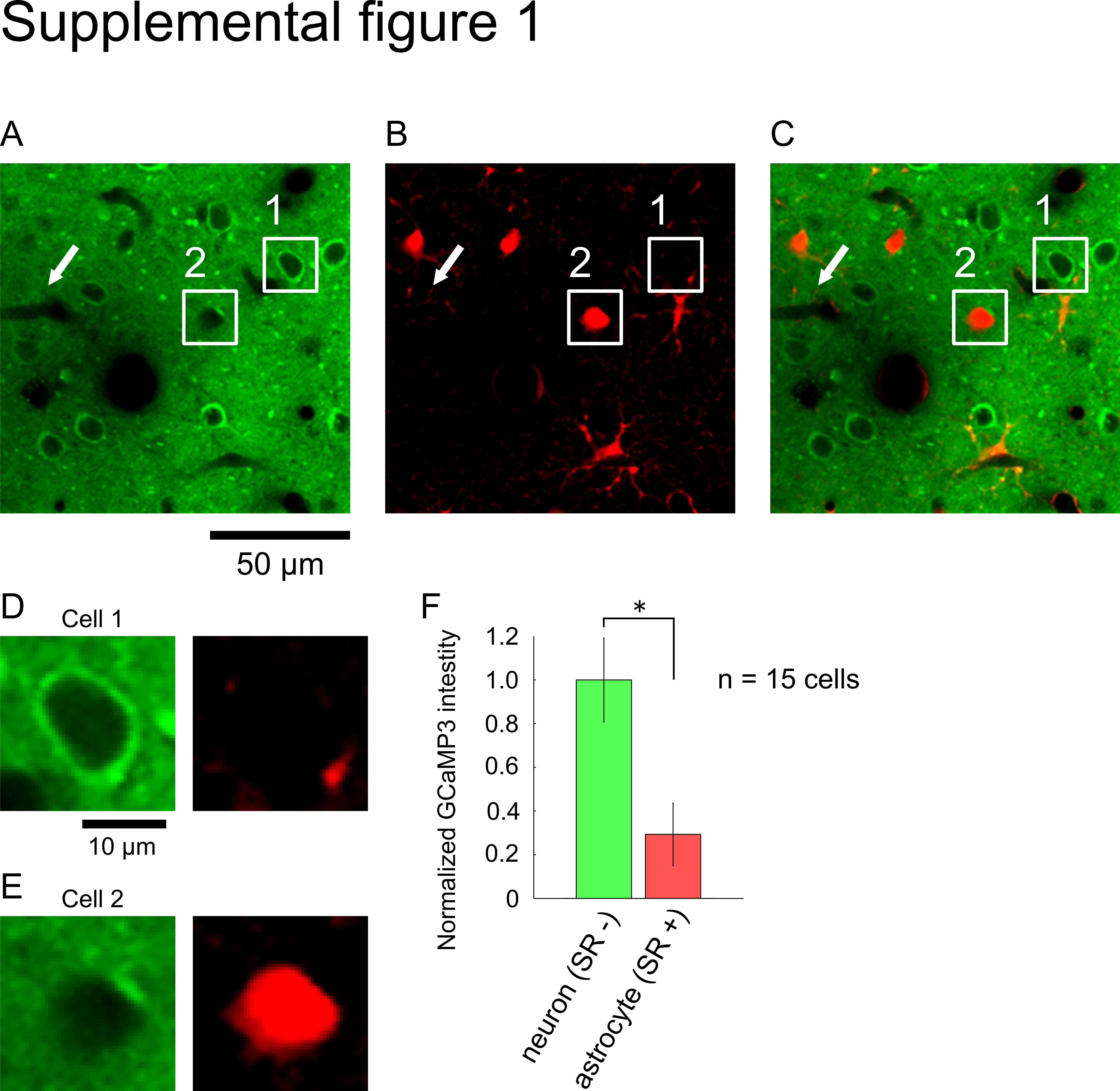

Supplement: Supplementary file 1 [file Image_1.JPEG]

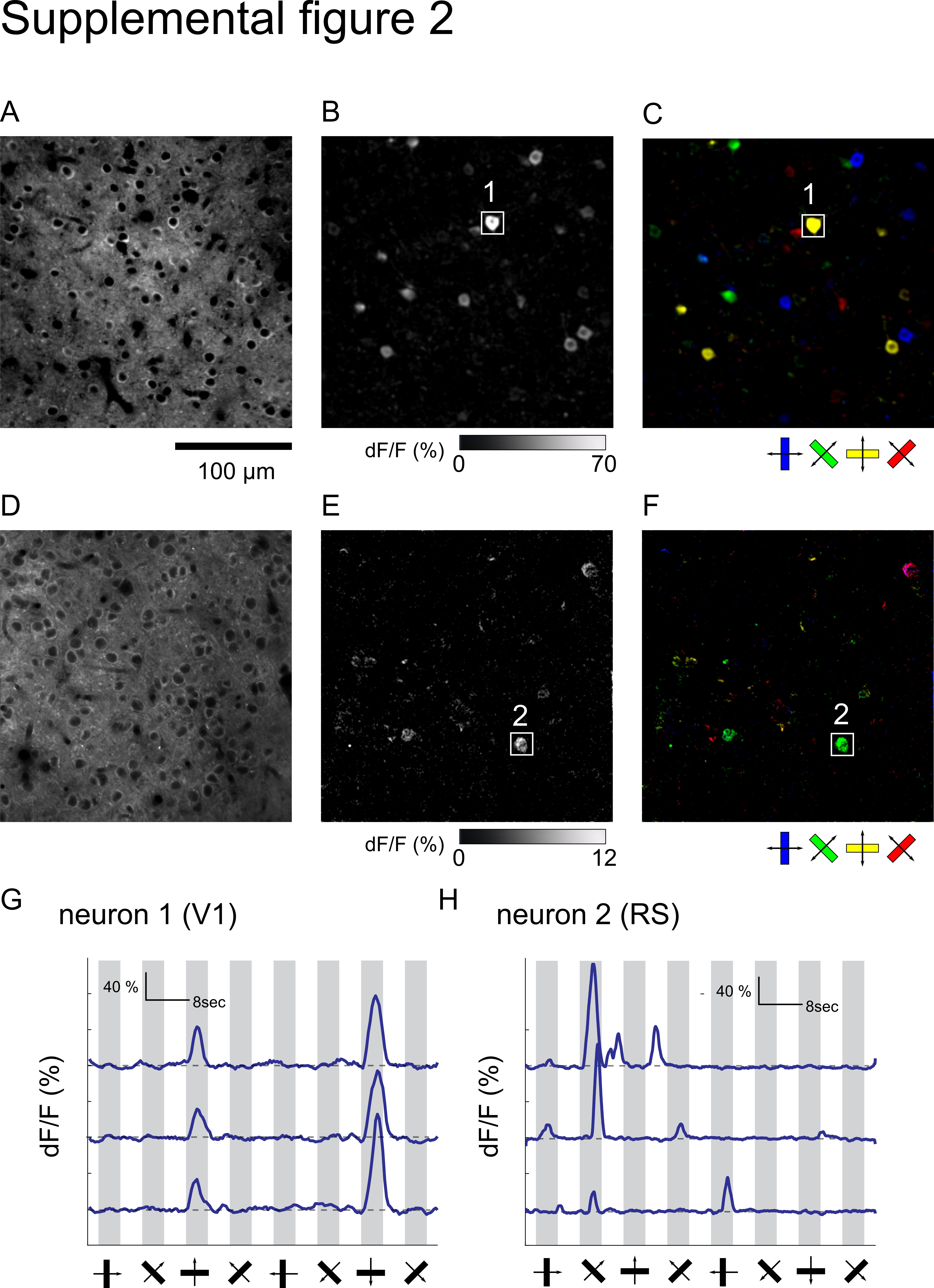

Supplement: Supplementary file 2 [file Image_2.JPEG]
